# Experimental Comparison of Biofidel Measuring Devices Used for the Validation of Collaborative Robotics Applications

**DOI:** 10.3390/ijerph192013657

**Published:** 2022-10-21

**Authors:** Jan Zimmermann, Michael Huelke, Matthias Clermont

**Affiliations:** Institute for Occupational Safety and Health of the German Social Accident Insurance (IFA), 53757 Sankt Augustin, Germany

**Keywords:** test method, mechanical hazards, collaborative robots, safety validation

## Abstract

Biofidel measuring devices are used to validate safety in collaborative workplaces. In these workplaces, humans work together with robots that are equipped with a Power and Force Limiting function (PFL). In this experimental comparison, differences between devices and possible causes are examined more closely. Safety-related parameters are identified in a literature review. Focusing on mechanical aspects, three biofidel measuring devices are analysed and compared in an experimental test series. To this end, a linear motor and a pendulum are used and the steps for comparing concepts are proposed and applied. Depending on the stiffness settings and the materials used, geometry effects on the force-deformation behaviour are shown. An oscillation occurred in one case. The comparison of the three devices shows average differences of 5% in measured peak force between them. This study helps to achieve uniform and comparable results in practice.

## 1. Introduction

This article presents a comparison of measuring devices used to validate safety in collaborative workplaces. In these workplaces, humans work together with robots that are equipped with a Power and Force Limiting function (PFL). In this way, the advantages of both humans and machines are combined, and more ergonomic work processes can be created. On the one hand, the focus has been on finding these limits with medical experiments on voluntary human subjects. On the other hand, measuring devices are developed to test if a robot meets these limits. Both activities have been driven, for more than 10 years, by the IFA working together with different partners from industry, research, and OSH organisations [1,2,3,4].

ISO TS 15066 contains preliminary biomechanical load limits for the assessment of collaborative robots that are protected by a power and force limiting safeguarding mode (EN ISO 10218-2) [5,6]. Approving these collaborative robot work cells requires measurement with a biofidel force-pressure measuring device that can measure the force curve as well as the pressure distribution and, at the same time, simulates the mechanical compliance of the human body [7]. The technical specification specifies typical deformations or stiffnesses for different body regions. However, no precise information is given on the measurement setup and possible measurement uncertainties. This can lead to different interpretations. It is already the case that existing force-pressure measuring instruments, applied with the same load, may achieve different measurement results. This could lead to a distortion of competition and counteract the protection objective.

In the standards, the description of the force measuring device is not very detailed. Only the German DGUV(German Social Accident Insurance) informative publication on collaborative robot systems offers some details about the device [8]. The document specifies two types of differences, which need to be considered according to the device itself and operation problems. Organisational gaps and practical measurement uncertainties due to the device configuration exist. For both types, important facts should be deduced, and test protocols implemented.

It must be ensured that the same measuring procedure is followed with all force-pressure measuring instruments from different manufacturers and that comparable measurement results are obtained under the same loads. In this paper, the most important measuring and instrument parameters are identified. and details for a uniform instrument specification are to be developed. The aim is to find methods that are able to compare different devices and test if relevant differences occur. This work is intended to support the establishment of an EU-wide measurement protocol for the testing of force- and power-limited robots, which will be carried out as part of the COVR toolkit (www.safearoundrobots.com (accessed on 31 August 2022)). COVR is shorthand for the EU-funded project “Being safe around collaborative and versatile robots in shared spaces”.

This paper presents a study of a comparison of three different measurement devices from different manufacturers with slightly different designs. The general architecture of the measuring device which all three devices are compared to in the paper follow, is described in Section 2 as well as the different experimental equipment and procedure to compare the functionality of the devices are presented. Section 3 gives examples of the measuring results. Finally, Section 4 presents an overall conclusion and main indications.

### Literature Review

The literature on robotics, in general, is very extensive. However, the safety aspects of robotics are only included rarely. In many cases, they are left out completely or are only considered very late in the design or implementation process. Some excerpts of the publications dealing with safety issues are reported here. Identified important parameters are marked with bold letters.

The general hazards for the human, related to robots, can be divided into three aspects: (1) the human is crushed by a pinch point between the robot and an obstacle, (2) the human is pushed into another dangerous machine by the robot, and (3) the human is injured by an impact with the robot or its payload [9]. Ulrich et al. (1995) identified sources of danger according to the assumption that injury involves a transfer of energy between a robot and a human. A fault tree analysis identified possible sources of danger.

Ulrich also described a matrix of combinations of possible injuries with possible effective safety mechanisms. Hence, some relevant parameters can be found like (1) static or impact **forces**, (2) static or impact **contact stress**, (3) **static or impact gripping or pinching forces**, (4) crushing forces and torques due to robot **weight** on a human, (5) static or impact torques, and (6) impact of a projectile released by the robot.

Based on this, a simplified list of safety specifications can be extracted: Static force, static gripping or pinching force, static contact stress, impact force, impact gripping or pinching force, impact contact stress, and crushing force from the robot weight. Each of these specifications should be minimised in a given design in order to maximise robot safety.

In the same way as Ulrich identified hazards and parameters for the human, he also looked at parameters for robot design. A system of robot functionality and performance metrics can be described using the following terms: (1) **agility**, (2) **strength**, (3) **position accuracy**, (4) **gentleness**, (5) **dexterity**, and (6) **lightness** [9]. Due to some assumptions and simplifications, the following list was generated: **end tip velocity, payload, position and force accuracy, dexterity, and lightness**. Therefore, to maximise the functionality and performance of a robot, each of these five specifications must be optimised.

The challenge is to design a robot for which all of the task specifications are maximised to deliver superior performance while all safety specifications are minimised to eliminate the risk of human endangerment. The balance between safety and product quality must be handled with care. Therefore, Ulrich recommends a safety diagram. This ends up with a methodology for a safe robot design consisting of six guidelines.

1. Maximise robot accuracy
2. Maximise robot dexterity
3. Minimise robot weight
4. Eliminate pinch points and maximise potential gripping radii
5. Maximise robot contact area
6. Maximise robot padding thickness

Compared to Ulrich’s very general approach, the one by Haddadin et al. (2011), who carries out many investigations on robot safety, is more specific with respect to the testing of robot safety. Different injury mechanisms are considered. Different scales evaluate different experiments, but it is difficult to derive a stringent solution approach from the large number of investigations. Haddadin compares human-robot collisions by scales like the Head Injury Criterion (HIC) or the Abbreviated Injury Scale (AIS), from road safety, which are used to avoid serious injuries and fatalities. He has noticed that these scales are often not very applicable to a working environment, because the expected injury severity is lower. But he gives a synopsis of possible injuries and relevant parameters [10].

The safety tree by Haddadin indicates possible injuries, relevant factors, expected injury severity, and possible indicators. It distinguishes between **quasi-static and dynamic loads and a clamped (constrained) and free (unconstrained) collision**. In addition, the proximity to a singularity configuration is mentioned. Haddadin discussed a comparison of safety scales and declared the need for a new low-ranking scale [10]. In addition to Ulrich (1995), the **contact situation and the shape of the tool or workpiece** should also be considered.

Oberer-Treitz (2018) derives two safety functions, which consider different parameters [11]. The main areas of focus were: the evaluation of the passive safety of robot systems; the identification of the load spectrum in robot collisions; the selection and development of suitable tools and models for estimating the collision potential of robot systems; the definition of general criteria for the evaluation of collision potential, and finally, the development of a method for the safety assessment of robot systems for the HRK. One of the results is a risk assessment function that is formed from various parameters. The collision potential must be less than a tolerable injury limit; this injury limit depends on the **body region** involved in the collision.

- The collision potential during **free impact (unconstrained)** depends on the following parameters: **effective mass in the collision, velocity at the collision point in the direction of the collision, radius of the colliding robot structure, and damping effects due to material or compliance** [11].
- The collision potential during **clamping (constrained)** depends on the following parameters: **acting force due to the active motors in the direction of the collision, velocity at the collision point in the direction of the collision, radius of the colliding robot structure, damping effects due to material or compliance, and maximum stop time (reaction and braking time)** [11].

For an injury potential estimation, the influencing parameters are **velocity, acceleration, geometry, material, location, environmental layout, accessibility for humans, and training level human activity injury criteria tolerance** [12].

The Health and Safety Executive (HSE) Research Report RR906 (2012) about collision and injury criteria when working with collaborative robots states that the collision forces that a robot might inflict can be influenced by [13]:

- The relative **velocity** at the moment of collision;
- The **shape, area, and hardness** of the contacting part;
- The **speed** of collision detection;
- The **mass** of the robot part, and how **quickly its movement/force can be braked**;
- The **software** programming of the robot.

Many of the mentioned parameters are also found in other publications, some briefer examples are presented in the following:

Mansfeld (2016 & 2018) distinguishes between impactor/robot parameters and subject/human impact parameters. The impactor/robot parameters are modelled in terms of **its instantaneous mass, velocity, curvature, and elastic surface properties**, while the subject is represented in terms of the **impact location, instantaneous mass, and velocity**. Therefore, this must be considered by the measuring device [14,15].

Vemula et al. (2018) show the influences **of robot mass and impact velocity, the robot’s radius of curvature and restitution coefficient,** and the **human’s stiffness properties** [16].

Matthias et al. (2011) again point out a safety diagram and take into account the typical steps of a risk analysis. Seven steps need to be fulfilled [17]:

(1) Limits of the machine
(2) Lifecycle phases
(3) Personnel involved
(4) Activities carried out
(5) Hazards encountered by the personnel involved
(6) Contact areas on the human body
(7) Injury scale to gauge the severity

Investigations made by Lim in 2000 show the behaviour of a passive, viscoelastic trunk, and passively movable base in response to different collision scenarios. However, in the experiments, the first peak force was practically the same for most scenarios, but the behaviour afterwards differed [18]. Additionally, Khatib et al. (1995) give a theoretical approach to the behaviour of the inertial properties in robotic manipulation [19].

As a model of human-robot collision, Ikuta et al. (2003) create a danger index alpha to compare different hazard risks: As an example, five indexes are represented as follows. They are represented by (1) reduce weight, (2) soft material, (3) joint flexibility, (4) shape, and (5) surface friction. In addition, three examples of safety control strategies are represented. These are (1) keeping distance, (2) approaching velocity, and (3) posture incl. (a) moment of inertia and (b) stiffness. With each of these danger indexes, robots can be compared. Furthermore, they are used to score weaknesses of a design and redesign parts or elements of a robot. The danger indexes contain at least the following parameters: **weight, velocity, force, area, contact time, moment of inertia, stiffness, displacement, joint angle, and friction coefficient** [20].

Finally, questions about the understanding of biofidelic behaviour, meaning biomechanical aspects, become more important. Huelke and Ottersbach (2012) focus on a concept of a force-pressure measuring system, which is described in the following part. The strategy of the biofidelic measuring instrument with the main biomechanical and measuring properties (**compression elements:** CC1 and CC2; software adaptation of the measurement signals to the **inertia and movement behaviour** of the colliding body region by correction functions: CFI and CFV) is shown, and the relevant limits and settings for the data acquisition are provided. Weight factors of the different device elements are not mentioned with regard to their impact. This might be a gap in research that requires further investigation. The actual settings that are suggested for use during a collision test are modified at a national level in the DGUV informative publication about collaborative robot systems [7,8].

Dagalakis et al. (2016) describe a dynamic impact testing and calibration instrument (DITCI). The testing instrument is a drop test with a spring-supported baseplate with a flexible foundation designed to simulate the flexible behaviour of a human body. A spring simulates **the stiffness of the human body**. Different tool tips are tested on synthetic skin tissue on top of ballistic gelatine to investigate the hazard of various loading conditions [21].

Kossmann (2019) focuses on the safety of the human-robot-interaction through a biofidelic valuation approach. His overview of influencing factors cites **velocity, mass, design of the tool, singularities, collision monitoring, body regions, and the identification of hazard points** [22]. Recently other studies using biofidel measuring devices to validate the safety of collaborative robot applications were found as well [23,24,25].

Since 2010, different research projects on the topic of human-robot collision have been carried out by Behrens. He has focused on the limits for pain pressure threshold and first low-level injuries for different body parts. Behrens distinguishes between **quasi-static and dynamic (transient) contact**, between **constrained and unconstrained situations,** and between **blunt and semi-sharp surfaces**. He investigates up to 28 different body parts as part of a volunteer study [1,2]. In terms of pain onset, the hand area is the most hardened to endure a collision. The risk of a low-level injury with a semi-sharp contact body was observed for body parts in the arm region and on the back of the hand. Therefore, in a transient contact situation, the behaviour of different body locations may be separated into regions with (a) thin skin on top of a bone and (b) muscle tissue regions.

## 2. Materials and Methods

### 2.1. Description of a Biofidel Measuring Device

**Figure 1:**
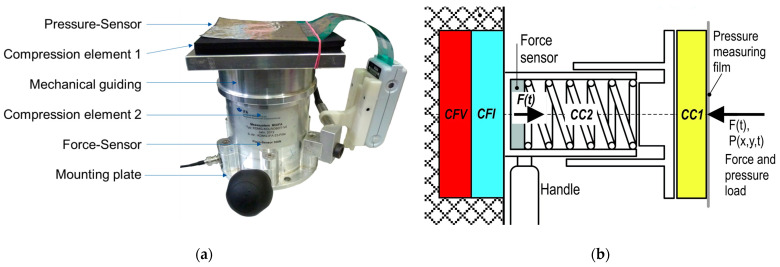
(**a**) Typical elements of a biofidel force-measuring device (prototype); (**b**) Schematic diagram of a biofidel force-measuring device.

The present section gives an overview of the biofidel force-measuring device architecture. Specifically, it presents important components and explains their functions. The functionality of a prototype device (Figure 1a) can be changed by manipulation of each component for specific evaluation of the impact on measuring results. The new findings are used for a final comparison to collision test devices that already exist on the market, whose architecture is comparable to the prototype device. Figure 1 shows the main functional components of the force-measuring device. The location of the compression elements (i.a., CC1 and CC2) and the force and pressure sensors are shown in the schematic diagram (Figure 1b).

The applied force and pressure are measured by a force sensor and pressure-measuring film. The two compression elements, a rubber or foam material (CC1) and an elastic mechanical spring (CC2)) ordered in a row, represent the characteristics of different body regions. Different versions of this device type with a measurement range of between 300 N to 1000 N are available on the market. The recording frequency of the implemented force sensor is at least 1 kHz, and the signal is filtered with a 100 Hz Butterworth low-pass filter. Different pressure-measuring systems, which can be time-discrete or are not available. The stiffnesses (CC2) for several body regions are given in ISO TS 15066 [5]. Information regarding CC1 is reported in national information on collaborative robots or is simply left out completely [8].

The force-measuring device of the described type has, for example, a flywheel mass of approximately 1.5 kg because of the mechanical guiding. The flywheel mass represents the freely moving mass of the measuring instrument, it is not the total mass. However, other devices have significantly lower weights.

The signal of the measured force-time curve can be separated into a dynamic behaviour and a quasi-static (clamping) situation [8]. The dynamic part is characterised by a peak force value due to the collision. After the dynamic collusion, a remaining clamping can occur. The clamped part is also called quasi-static, because the force remains constant over time. This research focuses on the dynamic behaviour.

To describe the hazard of the geometry, blunt and semi-sharp contours can be distinguished. For a blunt impact, the collision area is large. Hence, the peak force limits the allowed impact. If the collision area is semi-sharp, a second safety mechanism is important. The specific contact pressure needs to be observed to avoid local trauma to the human skin [1,2,3,4,26,27]. However, the influences of the pressure measuring equipment are not part of this investigation. For very small collision areas or sharp edges, stabbing and cutting injuries are a risk and must be avoided.

Three biofidel measuring devices which were available on the market were used for the study. For compliance reasons, the manufacturers were treated anonymously. The experimental equipment and procedure are presented here.

### 2.2. Experimental Equipment

**Figure 2:**
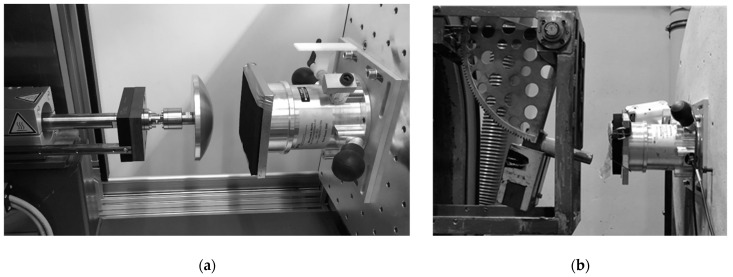
Example of the set up in the test benches: (**a**) Linear motor (left) of the test machine with a tappet and (**b**) pendulum.

Two test benches were available for tests and were used for the experiments at IFA. The test benches, which focus on examining the behaviour of force-measuring devices, are shown in Figure 2:

- A test machine (Figure 2a) executes linear quasi-static or dynamic movements of a tappet. The tappet is positioned at a defined distance from the measuring device. Using defined pulses, influences of different device parameters can be obtained.
- A pendulum (Figure 2b) is used to create a short impact to be applied on the measuring device with a defined and limited energy input.

#### 2.2.1. Test Machine

A customised test machine has been designed and manufactured by the IFA. The machine is comparable to the motion of a simple linear robot arm (1DOF) and tests the accuracy of the force-pressure measurement device under dynamic conditions. To this end, a powerful linear motor applies dynamic pulses to force-pressure measuring devices.

The stationary linear motor (LinMot) has a positioning accuracy of four-hundredth of a mm and a maximum holding force of 1 kN. With the use of a servo controller (drive) and an associated software program (LinMot-Talk) the motor device is controlled and parameterised. A customised program in LabView is used on the software side for data acquisition. The stationary position of the motor, which results from mounting the machine unit on a rigid construction made of square profiles, rules out measurement uncertainties caused by the deformation of the statics. The force-pressure measurement devices are fixed to a mounting plate positioned in the direction of travel of the linear motor. The mounting plate is also attached to the rigid construction and allows a fast exchange of the tested measurement devices. It is adjustable for different devices and settings.

To enable the pulses to be evaluated and feedback to be transmitted to the drive, a piezoelectric sensor is inserted in between the linear motor and its front end, where tappets are attached. To determine and evaluate its position, the linear motor device simulates an incremental path signal through the drive. The tappets differ in terms of their shape and geometry and can be changed.

During the test session, a movable guard controls the danger zone between the actor and the force-pressure measurement device, in order to prevent any dangerous collisions. Corresponding safety control modules are installed for this purpose.

**Figure 3:**
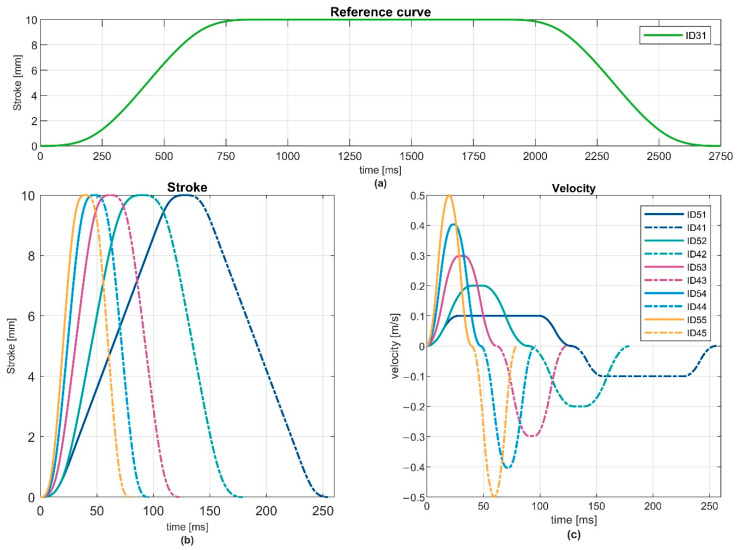
Examples of characteristic motion curves: reference curve with ID31 (**a**), stroke (**b**) and velocity (**c**) of the ten IDs used.

The linear forward and backward movement of the tappet is described by different characteristic curves (Figure 3). Forward movement is characterised by ID51–55, and forward plus backward movements are characterised by ID41–45. The curves apply mechanical path-regulated pulses in form of a step function or Dirac delta function. This was based on the consideration that mechanical implementation of the functions is not fully identical to the ideal behaviour, as seen in Figure 3. The curves are parameterised at the beginning of each measurement. The light green curve with ID31 is used as a reference curve. The movement represented by that curve is slower than the ones used for the test (ID41 to ID55). In the reference case, the tappet moves 10 mm in less than one second, then stays in place for about a second, and finally moves back to the pre-set home position. The home position is set to, e.g., 3 mm in front of the measurement device. To check the right device position, a peak force of 200 N should be reached when applying the reference curve. This is defined as a working point since, for many applications, the limit values are between 100 N and 300 N.

The bottom row of Figure 3 shows exemplary curves representing a stroke of 10 mm, which differ regarding the maximum velocity. The solid part of each curve represents a movement in one direction until the parameterised stroke is reached, while the dashed part also describes the reverse motion to the pre-set home position. The curves are triggered by an ID Number, which represents the general progression characteristics.

#### 2.2.2. Pendulum

A pendulum test has been designed and manufactured by the IFA and used in testing and certification for more than 40 years. The force-pressure measurement device can be loaded with a known energy. At each swing position, the energy of the system is described either by potential or by kinetic energy. According to the given deflection, the potential energy is assigned to a specific energy level, which is constant in terms of energy conservation. At the vertex, the kinetic energy is maximal.

The current energy of the force-pressure device is limited by the components, which are described as a mass-spring (-damper) system. With the known energy of the pendulum at the impact position recorded by the measurement device, it is possible to estimate the energy transmission and a possible loss of energy due to the stiffness of the device and the resulting damping behaviour.

The potential energy of the pendulum changes with altering notch positions (step number) determines the pendulum deflection. By adding an additional mass to the original pendulum, it is possible to increase the potential energy for a specific deflection value. Two energy levels are used for the test: 0.5 joules and 1 joule. The exact value deviates by up to five percent, but these were the closest combinations with sufficient accuracy for the comparing tests. The lower value was chosen because, in the hand and arm region, a lot of applications can work until this level, while the higher value already has a low severity in semi-sharp contacts [1,28,29].

The contact area of the pendulum is almost planar and rigid. In the contact position, the pendulum is exactly at the vertex position, and the tappet area is parallel to the force-pressure-measurement device. The measurement device is placed in a horizontal position to match that condition.

### 2.3. Experimental Procedure

The test stands were used to carry out a parameter analysis and a comparison study. A detailed description and results are given in the following section.

#### 2.3.1. Analysed Parameters

This study focused on the influence of parameters that are assigned to the mechanical properties of the measuring instruments, which were determined in the literature study. These are mainly the compression elements (CC1 and CC2), as well as different contact geometries. The characteristics of the different force-measuring components are shown, as well as the oscillation behaviour. Different stiffnesses and flywheel masses are tested with the pendulum. In addition, the effects of the mass of the moving parts of the system (flywheel mass) and the fixation of the systems to a structure are investigated, shown in Appendix A and Appendix B. The results of the parameter analysis will be shown together with the different elements of the comparison study, which followed the concept for a comparison protocol, described in the next section.

#### 2.3.2. Concept for a Comparison Protocol

To compare the results measured with different force-measuring devices, it is important to know the specific parameters of each element. For the mechanical elements, the characteristics of the compression element and the mass of the moving parts are particularly relevant. These should be recorded and compared. For an approximate assessment of the system, a path-regulated step function or Dirac delta function can be applied, whereby different velocities are taken into account. Additionally, the overall behaviour can be compared by applying comparable impacts.

For a comparison of the different devices, the testing procedure concept is carried out. The following elements are used:

- Comparisons of the different elements:
  ◦ The compression element 1 (CC1) were compressed up to 400 N with two different tappets. A flat circular tappet with rounded edges and a diameter of 50 mm and a spherical tappet with a radius of 25 mm.
  ◦ The compression element 2 (CC2) is compressed over the total range of the measuring device so that the spring stiffness can be compared.
  ◦ Finally, the combined stiffness of CC1 and CC2 was measured with the spherical tappet.
  ◦ If possible, the weight of the different moving parts (the flywheel mass) should also be measured.
- The dynamic behaviour can be recorded with the test machine. For this, the static value of 200 N was chosen as a reference point, and the starting point was placed 10 mm in front of it, as described earlier. Then, curves with different velocities were used to compare the peak forces and oscillation behaviour.
- For the final test, the pendulum was used. Different load levels were used to enable a comparison of the different devices in combination with the corresponding elements. Each setting was repeated 5 times and the mean value was determined.

These tests shall serve as an indicator as to whether the results of the devices are comparable and what differences, e.g., measurement uncertainties, may occur.

## 3. Results and Discussion

The results of the literature study are briefly summarised before the results of the experimental study are presented and discussed.

### 3.1. Parameters Identified in the Literature

A summary of the main important parameters from the literature review is given in Table 1. The parameters are separated into two groups, one group for robot parameters and one group for measuring device parameters. However, there could be more and also some related to the interaction of both, like alignment. In this study, the listed parameters marked with a * are investigated further.

### 3.2. Comparative Study with the Influence of Different Parameters

The comparison protocol is performed with three force-measuring devices. In the following parts, the results are presented, compared, and discussed. To guarantee objectivity, the manufacturers of the tested devices are not mentioned.

For this paper, two working points (Q-points) are investigated. Q-point 1 is a configuration with a SH70A-element (7-mm-thick layer with a shore hardness A of 70) for CC1 and a 40 N/mm spring as the CC2 element, while Q-point 2 uses a stiffer spring with 75 N/mm. In some tests, SH30A (14 mm) and SH10A (21 mm) materials were used in addition to the material mentioned above.

#### 3.2.1. Comparison of the Different Elements

**Figure 4:**
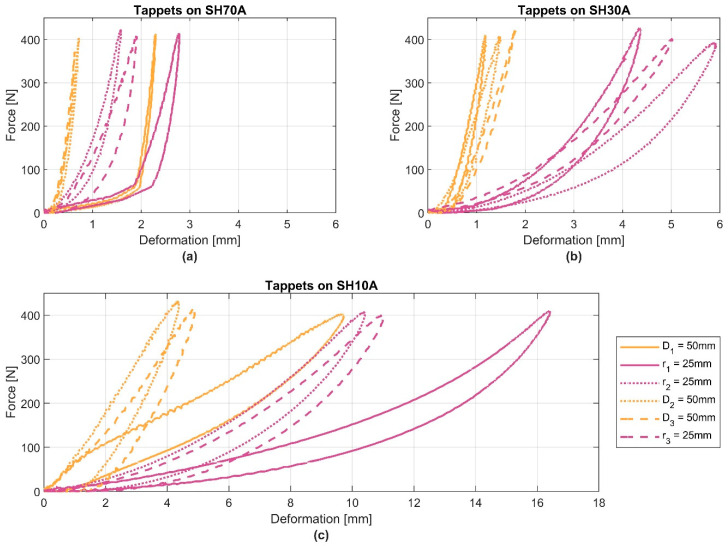
Force-deformation curves of three different CC1 materials (**a**) SH70A (**b**) SH30A and (**c**) SH10A from device 1 (continuous line), device 2 (dotted lines) and device 3 (dashed lines). Tappet geometry: spherical with radius (r) of 25 mm (purple) and flat with a diameter (D) of 50 mm (orange). Each device is marked with subscript indexes 1, 2 and 3.

In the following section, the procedure for the comparison protocol concept is applied to the three different devices. For the three different force measuring devices, three CC1 materials SH70A, SH30A, and SH10A, are tested with regard to their stiffness under load by using two different tappet geometries: a spherical tappet with a radius (r) of 25 mm and a flat tappet with a diameter (D) of 50 mm. Figure 4 shows the force-deformation curve for the different combinations.

All curves have hysteresis, which increases with smaller geometries and softer materials. The total deformation with the spherical tappet was greater than with the flat tappet. The principal behaviour of the force-deformation curves seems similar, but for device 1, a 2-mm shift of the SH70A material under low loads is visible. Also, for the SH10A element, the stiffness of device 1 is lower. Reasons for this might be the differences in material characteristics and ageing effects. For SH10A, a clear difference between device 1 and the other two devices was noticed. The SH10A element consists of a compliant foam, while for devices 2 and 3, it consists of a rubber material. It can be noticed that a shore value is basically an expression of the surface texture. It is questionable whether this information alone sufficiently differentiates technical requirements or whether further information is necessary to achieve clear comparability. However, especially for the less stiff materials, the geometry of the tappet has a clear effect on the curve characteristics; this is expected for human tissue as well. Also, for softer materials, velocity-dependent effects are expected. For comparability, it would be better if these can be avoided, e.g., by using less strain rate-dependent combinations.

**Figure 5:**
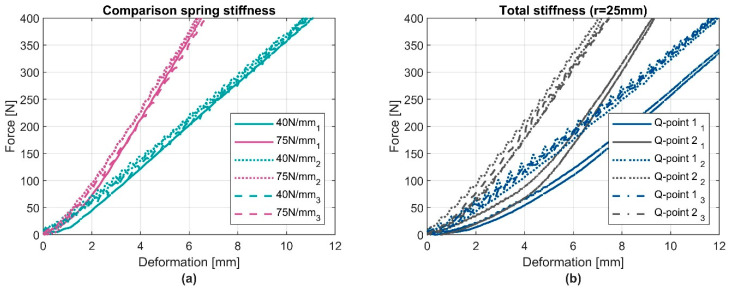
Comparison of spring stiffness (**a**) and the total stiffness (**b**) for the three devices respectively with configuration of Q-point 1 and 2. Tappet geometry: spherical with radius (r) of 25 mm. Each device is marked with subscript indexes 1, 2, and 3.

Differences in CC2 become apparent in a little shift at the beginning under small loads. Afterwards, the stiffness behaves in a similar manner and demonstrates nearly parallel progression. The explicit spring stiffnesses in both Q-points, (defined in Section 3.2) (Figure 5a) and the total stiffness (Figure 5b) are shown in Figure 5.

The three different devices are loaded with the same tappet (r = 25 mm). The noticeable shift (about 2 mm) in device 1, compared to the others, has also been observed with the CC1 element. It might come from a small bending of the supporting plate of the CC1 element itself. However, shifts under low forces are usually not noticed by the safety controls of a robot system, so they are less interesting as long the stiffness progression is parallel after this point.

In general, it is noticed that the mass of the moving parts (flywheel mass) of the measuring instrument ranges from a few 100 g up to 2 kg for the different measuring instruments on the market. Devices 2 and 3 had comparable low masses, while device 1 had a higher flywheel mass of approximately 1.5 kg.

#### 3.2.2. Comparison on the Test Machine

In the following section, the oscillation behaviour is evaluated for the three devices. For device 1 and Q-point 1, measurements with ID43 were repeated 30 times. The standard deviation of the maximum force was less than 1%, so only one measurement was taken for the descriptive description of the test series.

**Figure 6:**
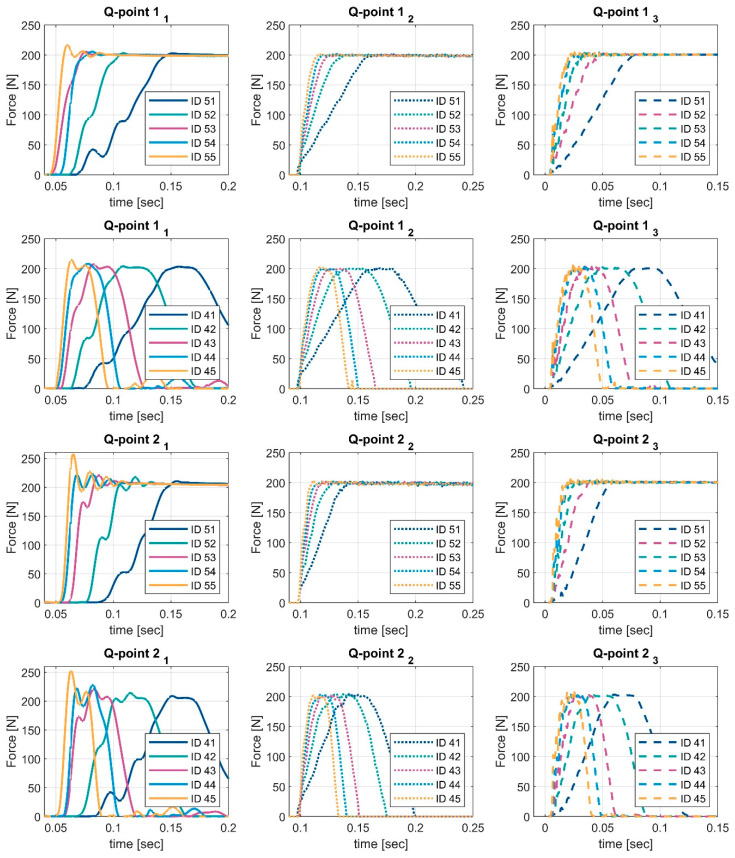
Measured force-time behaviour for devices 1 (**left**), 2 (**middle**), and 3 (**right**), when adding both Q-Point conditions under both loading movements ID 41–45 and ID51–55. Each device is marked with subscript indexes 1, 2 and 3.

The results of a test series, along with the different curve IDs, which are described in a previous chapter, are shown in Figure 6.

For the lower velocities (e.g., ID41, ID51), a very slight overshoot is visible for device 1. Additionally, small shifts are noticed in the rising part of the graph. The faster the curve velocity, the higher the force value when the shift occurs. When applying faster velocities (e.g., ID45) on device 1, the shift finally disappears, and the overswinging increases. Reasons for these shifts can be the acceleration and deceleration of the curves as well as the swinging behaviour of the compression materials, in addition to the mass and damping of the force-measuring device itself. For a stiffer configuration (Q-point 2), the overswinging behaviour increases. It should be noted that the test machine moves the full stroke in both velocity cases.

However, for the faster curves, measuring device 1 starts to swing, like a mass oscillator, and the peak values overshoot by about 10 to 25% compared to the quasi-static force values. This effect depends on the moving mass and the bearing, which means that the overswinging can also be smaller. This is indicated by the comparison with devices 2 and 3. This behaviour can also be affected by the CC1 element material.

When comparing the results of devices 2 and 3 with device 1, the results show that the overshoot is noticeably smaller. This is caused by a lower inertia for device 2 and device 3. The effect can be seen for both Q-points. The overshoot measured with device 2 and device 3 increases when a softer material is used for the CC1 element. Overshooting is not a danger, but it lowers the efficiency of a robot, so it should be avoided in order to achieve the maximum safe reduced speed of the applications. Furthermore, based on measurements with device 1 using a robot, the effect can be minimised through other control settings.

#### 3.2.3. Comparison with Pendulum Test

All devices are placed in the pendulum test bench. Devices are loaded with defined energy values to create comparability. Each value represents the mean value of 5 measurements, the percentual standard deviation of device 1 was an average of 1.4%, for device 2, the average was 0.4%, and device 3 had an average of 0.6%. The individual standard deviation shows that devices 2 and 3 are more stable compared to device 1.

**Figure 7:**
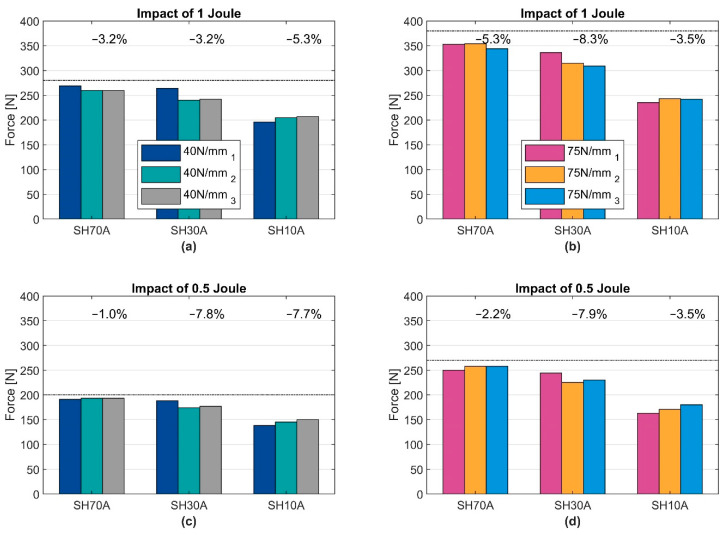
Measured mean force under two load levels [0.5 J (**c**,**d**) and 1 J (**a**,**b**)]. Comparison of CC1 elements combined with 40 N/mm (**a**,**c**) and 75 N/mm (**b**,**d**). Each device is marked with subscript indexes 1, 2 and 3. CC1 elements with a shore hardness of 70, 30 and 10 are used.

Figure 7 shows the peak force values for different CC1 elements and both CC2 elements of Q-point 1 and 2. The devices are loaded with an energy value of 0.5 joule and 1 joule. The black dotted line in the diagrams indicates the ideal force resulting from the spring-stiffness. Above each group, the percentage difference from the largest to the smallest value is shown.

The individual CC1 elements, which are used for the specific devices, differ in terms of their thickness and hardness. Generally, the softer and thicker the layer, the lower the maximum forces, as the results with SH10A show. The deviation of the measured minimal and maximal peak force varies by about 5%. The results show that deviation increases with softer CC1 elements (SH30A, SH10A). Especially in semi-sharp contact, the behaviour is more influenced by the soft tissue in the body regions. Due to low data availability, it is not possible to verify whether the biomechanical properties are sufficiently represented yet. So far, geometry influences have only been considered to a limited extent. Whereas the total behaviour of the device, when hard CC1 elements are used, is dominated by the CC2 elements. A hard CC1 element represents a body region with a bony substructure covered by thin, soft tissue structures. The differences show that a statement regarding whether the measured force is in an acceptable range is only valid if the correct stiffness has been considered and documented. Especially when a top layer is used, both compression elements behave like a series connection, which results in the real total stiffness of the device decreasing, as shown earlier. In order to achieve high comparability, it can be useful to select only one hard layer with low geometric influences.

Within the three devices used, when using the same settings, differences in peak force of up to 10 % are evaluated based on comparative measurements. The vibration behaviour, in particular, is significantly influenced by the overall arrangement of the compression elements and the flywheel masses. A system analysis will show the relationship more closely. A small comparative measurement (pre-study) with a robot shows that a difference of 5% was easily achieved under identical conditions. Due to the reactions of the robot, these differences may change with other equipment characteristics, e.g., especially with other CC1 compression elements or a different flywheel mass. Overall, a dynamic calibration of the measuring instruments and a corresponding measuring standard shall be considered.

All tests performed in this study only represent a small sample to test the methodology of the comparison protocol and are, therefore, not to be considered complete and exhaustive.

## 4. Summary and Conclusions

When robots assist human work, they often run a Power and Force Limiting safeguarding mode. So far, the only option to check if the force limits are complied with is a biofidel measuring device. Measuring devices are used to evaluate the loads that occur in a collision between a robot and a human. Tests with different measuring devices from different corporations show varying results, although the functional principle of the devices is the same. Differences in mechanical or electrical parameters of the specific device may lead to different measurement results. In this work, the effects of different mechanical parameters are highlighted, and an experimental comparison of different devices is carried out.

In a literature study, the relevant parameters in a collision situation were identified. They are divided into robot parameters and measuring device parameters, which are described by the functional architecture. In addition, a distinction can be made between mechanical and electrical aspects. In this study, the main mechanical aspects of a force pressure device used for collision testing are investigated with respect to their behaviour. The main influences of the compression elements (CC1 & CC2), the flywheel mass, and also the fixation of the measuring device were considered. Different geometries were considered, as well as different test curves with different load velocities. A self-designed linear testing machine and a calibrated pendulum were used for the tests. In the last step, a concept for a comparison test series was developed and performed with three force-measuring devices.

With regard to the behaviour of the first compression elements (CC1), the thickness, hardness, geometry of the contact body, and contact velocity can influence the results. For the second compression element (CC2), it is noticed that the behaviour corresponds to the expected relationship between energy, force, and spring stiffness. As long as the stiffness is linear and the overall behaviour is dominated by the CC2 element, it is important that the two elements together represent the correct biofidel properties. So far, the flywheel mass has not been considered a major factor in the existing literature, but the results show that the flywheel mass can influence the measuring results. Furthermore, the fixation situation affects the measured force values. Finally, a comparison of the three devices showed average deviations of about 5% in the measured peak force.

As far as we know, this is the first study that has created and applied such a systematic comparative approach. The comparison concept makes it possible to evaluate potential weaknesses further in order to finally develop a standard that enables measurements that are as comparable as possible. As a first step, this study should assist the performance of easy and verifiable measurements. In the long run, the knowledge of the exact measuring device characteristics can help to digitise them, so that, by using a model, an estimation can be calculated as early as the planning phase of an application.

The following indications can be derived from the examinations: Operators need to pay attention to the fixation of the measuring device and the rigidity of its structure and ensure that it uses the right combination of elements. The characteristics of the combinations used need to be documented to ensure the validity of the safety test achieved (e.g., the COVR protocols can be helpful [30]). Manufacturers must ensure that the correct spring stiffness is delivered and that the oscillation behaviour is known and transparent for each model. It is particularly important to document the flywheel mass in the technical data. In order to calibrate the measuring instrument correctly under dynamic loads, a defined impact must be applied to be able to assess the behaviour of the measuring instrument correctly. Therefore, the necessary information should be presented in a standard document. One factor in particular that has yet to receive sufficient consideration is the influence of geometries on biofidelic behaviour. This information should be scientifically prepared, discussed, and made known in the community.

## Figures and Tables

**Table 1:** List of identified main technical parameters.

Robot Parameters	Measuring Device Parameters
Velocity	Stiffness *
Effective mass	Effective (flywheel) mass (*)
Geometry, shape, area (*)	Filtering (data acquisition)
Stiffness, material	Resolution
(control) Force	Contact situation
Reaction + stopping time	Correction function
Inertia	Mounting (*)
… ^1^	… ^1^

^1^ Non-exhaustive list; * Considered in this article.

## Data Availability

Not applicable.

## Appendix A. Aspects of Fixation

The mechanical compliance of the force-measuring device depends on the fixation to the rigid test construction. For a test, with only one device, it is possible to make the fixation completely rigid by using screws or to have a more flexible suspension by using supporting springs between the base of the device and the mounting plat. The mechanical compliance is limited by the spring constants used. The effect of both methods is evaluated in this section. All springs are arranged in a parallel order, which leads to a summation of the spring constant value. Hence, increasing the number of springs used as the force-measuring device suspension leads to a higher stiffness and lower mechanical compliance. Arrangements of three, six, nine and twelve springs with spring constants of either 45 N/mm or 100 N/mm are used.

The possible effect is analysed for illustrative purposes for Q-point 1 and Q-point 2. To apply force, the test machine is used (linear motor). For the reference ID31, the force range is set to 200 N (Q-point 1) and 300 N (Q-point 2), then ID 43 is used for a dynamic test. In the fixed configuration, the peak value for the more dynamic impact therefore increases, as expected. Results are shown in Table A1.

**Table A1:** Percentage decrease in the event of a yielding fixation.

	REF	Fixed	Number of Supporting Springs			
			12x	9x	6x	3x
Test Machine						
Q-point 1 on 45 N/mm springs	200 N (static)	205 N (dyn.)	−2.4%	−2.9%	−3.4%	−10.7%
Q-point 1 on 100 N/mm springs	200 N (static)	211 N (dyn.)	−1.6%	−1.9%	−4.7%	−18.7%
Q-point 2 on 45 N/mm springs	300 N (static)	321 N (dyn.)	−7.6%	−8.1%	−9.5%	−15.2%
Q-point 2 on 100 N/mm springs	300 N (static)	314 N (dyn.)	−5.1%	−6.4%	−9.2%	−18.2%
Pendulum						
Q-point 1 on 45 N/mm springs	1 joule	268 N (dyn.)	−0.7%	−1.1%	−1.5%	−6.3%
Q-point 2 on 45 N/mm springs	1 joule	357 N (dyn.)	−3.1%	−3.6%	−5.9%	−18.2%

REF = reference, dyn. = dynamic.

The maximum value is reached in the fixed configuration, while the lowest spring number tends to result in distinct loss. The effect seems greater for higher measurement device stiffnesses, whereas lower stiffnesses would be less sensitive to the mounting rigidity. However, it can be expected that a measuring device mounted on a flexible structure will easily lead to 5% to 10% lower values. It is recommended to always mount the measuring device as rigidly as possible to the test stand.

For all configurations, a jump between the fixed and the compliant mechanism is observed. Particularly high mechanical compliance leads to a strong decrease of the force peak and should be avoided. Variants represented by 12x or 9x supporting springs seem typical for configurations, as they can occur in mobile test setups, e.g., with aluminium profiles. Compared to the fixed measurements, errors of up to 10% in a dynamic impact are to be expected if the operator does not pay attention to the rigidity of their structure. Larger deviations should be observed with the naked eye in the form of a movement of the measuring device base and can thus be directly detected and avoided.

Since the test machine is pathway-controlled, it is to be expected that, with an actively controlled robot or a defined energy input, the magnitude of the effect may change.

For testing of the fixation, the pendulum is used in combination with a defined load level input. In the two bottom lines of Table A1, the results of an impact while the measuring device is mounted on 45 N/mm springs (12x, 9x, 6x or 3x parallel) compared to the results for a fixed configuration are shown for the two Q-points.

Compared to the fixed configuration, the measured force decreases with decreasing stiffness of the construction. The lowest variant showed a significant decrease, and a movement of the base was also visibly detected in this configuration, while for the other flexible configuration, the movement of the base may not be seen by the inspector. For a stiffer collision configuration and higher loads, it seems even more important to have a stiff base contraction. In summary, rigid fixation of the measurement device is needed. If a movement of the device is noticeable after contact, the results are not reliable in any case.

## Appendix B. Flywheel Mass

The flywheel mass of the device influences the measurement results, especially in dynamic situations. Hence, it is necessary to know the influence when comparing measuring devices that differ in terms of their shape, size and mass. Additional flywheel masses were mounted to a test device to change the flywheel mass of the moving parts of the test device.

Table A2 gives an example for the percentage change. Here, the peak force decreases with increasing free-moving mass. However, this effect is dependent on the position of the flywheel mass and also depends on the combination of compression elements. The load level also has to be considered.

**Table A2:** Example of percentage change of measured peak force due to altered flywheel masses at an impact of 1 joule.

Extra Mass	Q-Point 1	Q-Point 2
Normal	277 N	369 N
+0.5 kg	−1.0%	−2.3%
+1 kg	−4.8%	−3.4%
+1.5 kg	−4.9%	−6.3%
+2 kg	−8.0%	−10.0%
+2.5 kg	−9.6%	−10.4%

Since, in practice, new devices are usually smaller and have lower flywheel masses, it is not expected that this effect has to be considered in particular in the future.
